# Obesity indices and diabetes risk among hypertensive patients: insights from the China Multi-Ethnicity Cohort study

**DOI:** 10.3389/fendo.2025.1518060

**Published:** 2025-03-10

**Authors:** Enhui Zhou, Feng Hong

**Affiliations:** School of Public Health, the Key Laboratory of Environmental Pollution Monitoring and Disease Control, Ministry of Education, Guizhou Medical University, Guiyang, China

**Keywords:** atherogenic index of plasma, obesity, diabetes, hypertension, minority

## Abstract

**Background:**

The atherogenic index of plasma (AIP) is recognized as a surrogate marker for dyslipidemia. It has been well-established that the AIP is significantly associated with diabetes, and obesity is a known risk factor for both dyslipidemia and diabetes. However, the relationship between obesity and diabetes, as well as the potential role of the AIP in hypertensive minority populations, remains unclear. This study aimed to assess the association between obesity index and diabetes in hypertensive people.

**Methods and results:**

This cross-sectional study included 9,446 participants from the China Multi-Ethnicity Cohort (CMEC) study. Our study suggested that obesity indices were significantly higher in diabetic patients compared to those without. Moreover, logistic regression analysis suggested that higher quartiles of obesity indices were associated with an increased risk of diabetes whether in crude or adjusted models (*p* < 0.05). Mediation analysis revealed that the association between obesity and the risk of diabetes, mediated by body mass index (BMI), waist-to-hip ratio (WHR), waist-to-height ratio (WHtR), and body adiposity index (BAI), through the AIP was 17.2%, 15.3%, 15.8%, and 19.2%, respectively. Additionally, restricted cubic spline analysis revealed a non-linear relationship between obesity indices and diabetes.

**Conclusion:**

In summary, obesity is significantly associated with diabetes in hypertensive minority Chinese, with the AIP partially mediating this relationship.

## 1 Introduction

Diabetes mellitus, specifically type 2 diabetes (T2DM), is more than just a global health issue; it serves as a precursor to a cascade of debilitating conditions (1). Numerous studies have demonstrated that hypertension is a significant predictor of the incidence of DM (2). The coexistence of these conditions, often referred to as the diabetes–hypertension syndrome, confers a significantly increased risk of cardiovascular events and reproductive health issues, thereby influencing patient morbidity and mortality (3, 4). Obesity, marked by excessive body fat accumulation, is a pivotal factor in the development of both hypertension and DM (5). Obesity’s complexity necessitates multifaceted assessment using metrics such as body mass index (BMI), waist-to-hip ratio (WHR), waist-to-height ratio (WHtR), and body adiposity index (BAI), each of which reveals distinct aspects of body fat distribution and associated health risks (6–9). Studies have demonstrated that obesity fuels insulin resistance, a hallmark of DM (10); it also exacerbates hypertension through mechanisms such as increased sympathetic nervous system activity, altered renal function, and changes in adipokine activity (11, 12). This relationship underscores the critical importance of weight management in hypertensive patients to mitigate the onset of diabetes. The atherogenic index of plasma (AIP) is an emerging biomarker that reflects the balance between pro-atherogenic and anti-atherogenic lipids (13). It has garnered attention for its potential in predicting DM risk, particularly in the context of atherogenic dyslipidemia, which is prevalent in obesity (14, 15). Abnormal AIP values indicate an imbalance in lipid metabolism and contribute to atherosclerosis, which subsequently leads to DM (16). The interrelationship among obesity, hypertension, and diabetes underscores the importance of understanding the mediating factors for effective prevention and management. In this context, understanding the role of the AIP as a mediator in the relationship between obesity and diabetes in hypertensive patients is particularly important. However, the precise role of the AIP in mediating the relationship between obesity indices and the risk of diabetes in hypertensive patients remains poorly understood. Such lipid metabolism abnormalities, driven by obesity, have been postulated to partially explain the increased risk of diabetes in hypertensive patients due to elevated AIP levels. Therefore, this study investigates the mediating role of the AIP in the relationship between obesity and diabetes risk in hypertensive patients.

## 2 Methods

### 2.1 Study participants

**Figure 1:**
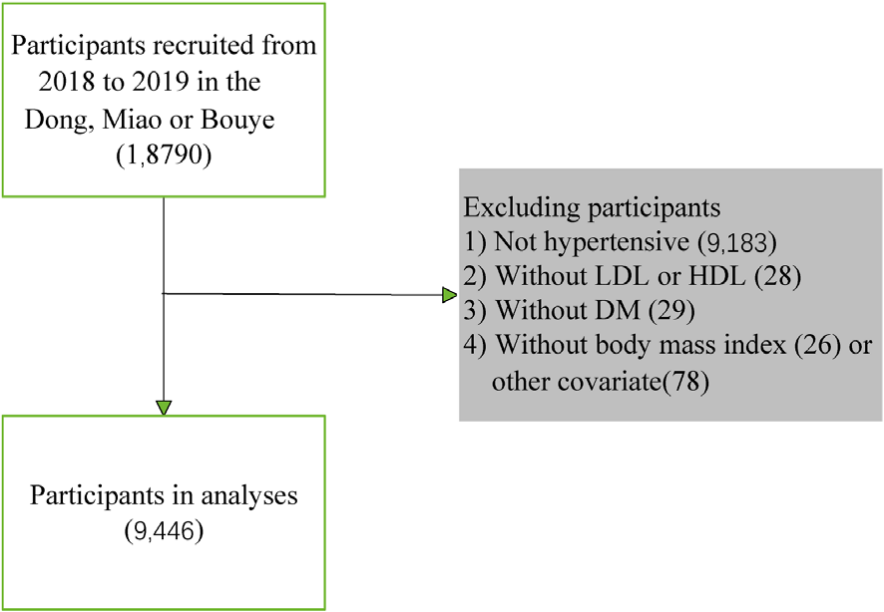
Flow diagram for the screening procedure.

Based on the China Multi-Ethnicity Cohort (CMEC) study, a multistage, stratified cluster sampling method was adopted in Guizhou Province as the study site to conduct a baseline survey among permanent residents aged 30–79 years from July 2018 to August 2019. Initially, a total of 18,790 people from the Miao, Dong, and Bouyei ethnicity groups were selected from the database. Furthermore, 9,183 non-hypertensive participants, 28 participants without high-density lipoprotein or low-density lipoprotein, 29 participants with incomplete diabetes mellitus data, and 26 participants with body mass index or 78 other incomplete covariate variables data were excluded. Finally, 9,446 participants were included in the analyses. The final study population consisted of 9,446 individuals (**Figure 1**). All participants signed informed consent forms before the investigation. This study was approved by the Sichuan University Medical Ethical Review Board (K2016038) and the Medical Ethics Committee of the Affiliated Hospital of Guizhou Medical University [2018(094)].

### 2.2 Clinical, anthropometric, and laboratory measurements

A questionnaire covering sociodemographic characteristics, medical history, family history, and lifestyle factors was used during an interview by the same group of trained experienced personnel. The CMEC collected participants’ blood samples after at least 8 h of fasting and measured levels of cholesterol (CHOL), triglyceride (TG), low-density lipoprotein (LDL-C), and high-density lipoprotein (HDL-C) by an AU5800 Automated Chemistry Analyzer (Beckman Coulter Commercial Enterprise, Shanghai, China).

### 2.3 Definition of variables

In order to include prehypertensive patients in this study, variables were defined as follows: hypertension was defined as systolic blood pressure ≥130 mmHg, diastolic blood pressure ≥80 mmHg (17, 18), or a self-reported previous diagnosis of hypertension. DM was defined using a previous diagnosis or glycosylated hemoglobin (HBA1C1) ≥6.5% or FPG ≥7.0 mmol/L. Hyperlipidemia was defined as a self-reported previous diagnosis of hyperlipidemia by a physician. The cardiovascular disease (CVD) outcome was defined as a previous diagnosis of coronary heart disease, stroke, or peripheral arterial disease and was recorded in the registration platform.

### 2.4 Anthropometric measures

Anthropometric measurements were conducted by well-trained nurses and physicians. Weight was assessed using an electronic scale with the patients in light clothes and without shoes. Participants were asked to stand with arms hanging freely and height was acquired by a stadiometer with the patient barefoot. BMI was calculated as weight (kg) divided by height squared (m^2^). Waist circumference (WC) was measured using an inelastic tape measure. Participants were asked to wear light clothes, and WC was assessed midway between the last rib and iliac crest with the participants breathing out gently. Duplicate measures were taken for all measurements with a tolerance error of 1 cm for height and circumference measurements and 1 kg for the weight measurement. A third measurement was needed if the difference of the first two measures was greater than the tolerance limit. The average of the two closest measurements was recorded at last. BAI was calculated as proposed by Bergman et al. (19): hip circumference (cm) divided by [height (m)]^1.5^ minus 18. WHR was calculated according to the following formula: WC (cm)/hip circumference (cm). WHtR was calculated according to the following formula: WC (cm)/height (cm). The AIP was calculated as the logarithm of the ratio of TG/HDL-C.

### 2.5 Covariates

In this study, the covariates included demographic characteristics (age, sex, and ethnicity), socioeconomic indicators (education level, household income, residence, and job type), physical activity, alcohol consumption status, smoking status, habit of drinking tea, systolic blood pressure, diastolic blood pressure, high-density lipoprotein, low-density lipoprotein, triglyceride, cholesterol, history of CVD, and hyperlipemia. Ethnicity was classified as Dong, Miao, and Bouyei. The participants’ education level was divided into three levels: lower than high school, high school or equivalent, and above high school. The job type was grouped into four categories: primary industry practitioner, secondary industry practitioner, tertiary industry practitioner, and unemployed or other. Household income per year was divided into three levels: <100,000 CNY, 100,000–199,999 CNY, and ≥200,000 CNY. Smoking condition was categorized into three groups: never, current, and ever. Alcohol consumption condition was categorized into three groups: never, occasionally, and frequently. Habit of drinking tea was categorized into two groups: yes and no. Physical activity was categorized into three groups: low, moderate, and high.

### 2.6 Statistical analysis

Continuous variables collected from the participants were tested for normality according to the characteristics of the data, and data obeying a normal distribution were expressed as mean ± standard deviation (SD), while data not obeying a normal distribution were expressed as median and interquartile range (IQR). Qualitative variables were expressed as relative numbers or percentages. Differences in characteristics between the DM and non-DM groups were evaluated using the Student’s *t*-test (for continuous variables) or chi-square test (for categorical variables). Differences between subjects grouped by quartiles of obesity index were compared in multivariable logistic regression, using quartile 1 (Q1) as the reference group, and the values of odds ratios (ORs) and 95% confidence intervals (CIs) were calculated. Three logistic models were developed for the analyses: model 1 was not adjusted for any confounding variables and was a univariate analysis; model 2 was adjusted for the main demographic variables (sex, age, and ethnicity); and model 3 added place of household registration, education level, job type, family income per year, smoking status, alcohol intake status, habit of drinking tea, physical activity, systolic blood pressure (SBP), diastolic blood pressure (DBP), HDL-CH, LDL-CH, TG, CHOL, CVD, and hyperlipemia to model 2. Linear regression was used for continuous variables. To visualize more closely the linear or non-linear correlation between obesity indices and DM, curves were fitted using restricted cubic spline (RCS). Finally, mediation analyses were performed using the mediation package, and confidence intervals for the mediating effect were assessed using the bootstrap method to determine the proportion of the mediating effect accounted for by the AIP. Using these statistical methods, the possible causal relationship between four obesity indices and DM can be examined more broadly. Statistical analyses were performed using the R software (version 4.2.0). Statistical significance was defined as *P <*0.05.

## 3 Results

### 3.1 Characteristics of the study population and correlation of obesity indicators

**Table 1** shows the baseline characteristics of the study participants. In the whole group of 9,446 hypertensive patients, there were 1,111 patients with diabetes, and the prevalence of diabetes was 11.76%. The medium age of the study population was 53.7 (46.8, 62.5) years for non-DM people and 56.4 (51.3, 65.3) years for DM people. In addition, the prevalence of DM was significantly different stratified by age, sex, ethnicity, place of household registration, education level, job type, family income per year, smoking status, alcohol intake status, habit of drinking tea, physical activity, SBP, DBP, HDL-CH, LDL-CH, TG, CHOL, CVD, and hyperlipemia. Furthermore, there were significant differences in ethnicity between the two groups (**Table 1**).

**Table 1:** Basic characteristics of the study participants.

Variables	Overall	Non-DM	DM	*p*-value
	9,446	8,335	1,111	
Age [median (IQR)]	54.1 (47.3, 62.9)	53.7 (46.8, 62.5)	56.4 (51.3, 65.3)	<0.001
Male (%)	3,978 (42.1)	3,413 (40.9)	565 (50.9)	<0.001
Ethnicity (%)				0.773
Dong	3,474 (36.8)	3,076 (36.9)	398 (35.8)	
Bouyei	3,142 (33.3)	2,765 (33.2)	377 (33.9)	
Miao	2,830 (30.0)	2,494 (29.9)	336 (30.2)	
Urban residence (%)	2,079 (22.0)	1,718 (20.6)	361 (32.6)	<0.001
Education level (%)				<0.001
Lower than high school	7,721 (81.7)	6,879 (82.5)	842 (75.8)	
High school or equivalent	860 (9.1)	729 (8.7)	131 (11.8)	
Above high school	865 (9.2)	727 (8.7)	138 (12.4)	
Job type (%)				<0.001
Primary industry practitioner	4,008 (42.5)	3,623 (43.5)	385 (34.7)	
Secondary industry practitioner	758 (8.0)	699 (8.4)	59 (5.3)	
Tertiary industry practitioner	3,134 (33.2)	2,736 (32.9)	398 (35.9)	
Unemployed or other	1,534 (16.3)	1,267 (15.2)	267 (24.1)	
Household income per year (%)				<0.001
<100,000 CNY	8,629 (91.5)	7,647 (91.9)	982 (88.5)	
100,000–199,999 CNY	717 (7.6)	608 (7.3)	109 (9.8)	
≥200,000 CNY	87 (0.9)	69 (0.8)	18 (1.6)	
Smoking (%)				<0.001
Never	7,155 (75.7)	6,375 (76.5)	780 (70.2)	
Current	1,826 (19.3)	1,571 (18.8)	255 (23.0)	
Ever	465 (4.9)	389 (4.7)	76 (6.8)	
Alcohol (%)				0.032
Never	4,579 (48.5)	4,001 (48.0)	578 (52.0)	
Occasionally	3,400 (36.0)	3,035 (36.4)	365 (32.9)	
Frequently	1,467 (15.5)	1,299 (15.6)	168 (15.1)	
Habit of drinking tea (%)	1,508 (16.0)	1,261 (15.1)	247 (22.2)	<0.001
Physical activity (%)				<0.001
Low	3,310 (35.7)	2,833 (34.7)	477 (43.9)	
Moderate	3,031 (32.7)	2,694 (33.0)	337 (31.0)	
High	2,918 (31.5)	2,646 (32.4)	272 (25.0)	
SBP [median (IQR)]	135.0 (125.3, 148.3)	134.7 (125.0, 147.7)	139.7 (129.7, 152.3)	<0.001
DBP [median (IQR)]	87.0 (82.3, 93.7)	86.7 (82.3, 93.3)	88.0 (83.0, 95.3)	<0.001
HDL_CH [median (IQR)]	56.1 (48.3, 65.0)	56.5 (48.7, 65.4)	53.8 (45.6, 61.5)	<0.001
LDL_CH [median (IQR)]	109.4 (88.6, 131.5)	108.3 (87.8, 130.7)	116.0 (93.2, 140.8)	<0.001
TG [median (IQR)]	141.7 (101.0, 211.7)	137.3 (99.2, 202.8)	184.2 (123.6, 284.3)	<0.001
CHOL [median (IQR)]	194.5 (171.7, 220.0)	193.4 (170.5, 218.5)	205.0 (179.4, 232.0)	<0.001
History of CVD (%)	385 (4.1)	318 (3.8)	67 (6.0)	0.001
Hyperlipemia (%)	410 (4.3)	279 (3.3)	131 (11.8)	<0.001
BAI [median (IQR)]	25.1 (22.4, 29.6)	25.6 (22.1, 29.2)	27.9 (24.6, 31.5)	<0.001
WHR [mean (SD)]	0.9 (0.1)	0.9 (0.1)	1.0 (0.1)	<0.001
WHtR [median (IQR)]	0.5 (0.5, 0.6)	0.5 (0.5, 0.6)	0.6 (0.5, 0.6)	<0.001
BMI [median (IQR)]	24.8 (22.6, 27.1)	24.6 (22.4, 26.9)	26.2 (24.0, 28.5)	<0.001
AIP [median (IQR)]	0.4 (0.2, 0.6)	0.4 (0.2, 0.6)	0.5 (0.4, 0.8)	<0.001

DM, diabetes mellitus; SBP, systolic blood pressure; DBP, diastolic blood pressure; HDL_CH, high-density lipoprotein: LDL_CH, low-density lipoprotein; TG, triglyceride; CHOL, cholesterol; CVD, cardiovascular disease; BAI, body adiposity index; WHR, waist-to-hip ratio; WHtR, waist-to-height ratio; BMI, body mass index; AIP, atherogenic index of plasma.

### 3.2 Association of obesity index and AIP with diabetes mellitus

Regarding subjects in the lowest quartile of each indicator, ORs (95% CIs) for those in the highest quartile were 4.961 (3.351, 7.345), 5.965 (3.825, 9.304), 5.903 (3.821, 9.121), 3.932 (2.725, 5.672), and 3.280 (2.331, 4.615) for BAI, WHR, WHtR, BMI, and AIP, respectively, after adjusting for all covariates (**Table 2**). The exposure–response trend of DM with five indicators was observed (all *p*-trend < 0.05). Every 0.1-unit increment of BAI, WHR, WHtR, and AIP was associated with a 30.5%, 95.8%, 118%, and 21.2% increased risk of DM, respectively; every 1-unit increment of BMI was associated with a 14.4% increased risk of DM.

**Table 2:** Association between adiposity indicators or AIP and diabetes mellitus.

Variable	Model 1	Model 2	Model 3
	OR (95% CI)	OR (95% CI)	OR (95% CI)
BAI			
Q1 (≤22.40)	1.00	1.00	1.00
Q2 (22.40–25.91)	1.956 (1.572, 2435)^***^	2.150 (1.723, 2.683)^***^	1.683 (1.104, 2.563)^*^
Q3 (25.91–29.60)	2.401 (1.940, 2.972)^***^	2.886 (2.321, 3.589)^***^	2.079 (1.383, 3.123)^***^
Q4 (>29.60)	3.782 (3.085, 4.635)^***^	5.210 (4.189, 6.481)^***^	4.028 (2.681, 6.052)^***^
*p* for trend	<0.001	<0.001	<0.001
Every 1-unit increment	1.085 (1.072, 1.097)^***^	1.110 (1.096, 1.124)^***^	1.088 (1.064, 1.112)^**^
WHR			
Q1 (≤0.87)	1.00	1.00	1.00
Q2 (0.88–0.92)	2.448 (1.894, 3.165)^***^	2.362 (1.825, 3.056)^***^	2.451 (1.512, 3.974)^***^
Q3 (0.93–0.97)	3.993 (3.129, 5.094)^***^	3.759 (2.943, 4.802)^***^	3.920 (2.482, 6.190)^***^
Q4 (>0.98)	7.006 (5.541, 8.858)^***^	6.415 (5.067, 8.122)^***^	5.965 (3.825, 9.304)^***^
*p* for trend	<0.001	<0.001	<0.001
Every 0.1-unit increment	2.461 (2.243, 2.701)^***^	2.366 (2.153, 2.600) ^***^	1.958 (1.653, 2.319) ^***^
WHtR			
Q1 (≤0.51)	1.00	1.00	1.00
Q2 (0.52–0.55)	2.209 (1.745, 2.797)^***^	2.389 (1.883, 3.030)^***^	2.685 (1.693, 4.257)^***^
Q3 (0.56–0.59)	3.286 (2.624, 4.116)^***^	3.638 (2.896, 4.569)^***^	3.676 (2.361, 5.724)^***^
Q4 (>0.60)	4.867 (3.914, 6.052)^***^	5.825 (4.652, 7.294)^***^	5.903 (3.821, 9.121)^***^
*p* for trend	<0.001	<0.001	<0.001
Every 0.1-unit increment	2.308 (2.087, 2.551) ^***^	2.607 (2.341, 2.902) ^***^	2.189 (1.813, 2.642)^***^
BMI			
Q1 (≤22.58)	1.00	1.00	1.00
Q2 (22.59–24.84)	1.332 (1.075, 1.650)^**^	1.474 (1.187, 1.831)^***^	1.641 (1.104, 2.441)^*^
Q3 (24.85–27.16)	2.067 (1.692, 2.525)^***^	2.390 (1.949, 2.931)^***^	2.528 (1.734, 3.688)^***^
Q4 (>27.17)	3.114 (2.572, 3.771)^***^	3.849 (3.159, 4.690)^***^	3.932 (2.725, 5.672)^***^
*p* for trend	<0.001	<0.001	<0.001
Every 1-unit increment	1.141 (1.121, 1.161)^***^	1.169 (1.147, 1.190)^***^	1.129 (1.093, 1.166)^***^
AIP			
Q1 (≤0.22)	1.00	1.00	1.00
Q2 (0.23–0.41)	1.453 (1.171, 1.803)^***^	1.462 (1.199, 1.818)^***^	1.423 (0.981, 2.064)
Q3 (0.42–0.62)	1.948 (1.585, 2.395)^***^	2.003 (1.626, 2.466)^***^	2.042 (1.440, 2.896)^***^
Q4 (>0.63)	3.556 (2.931, 4.314)^***^	3.884 (3.189, 4.731)^***^	3.280 (2.331, 4.615)^***^
*p* for trend	<0.001	<0.001	<0.001
Every 0.1-unit increment	1.155 (1.133, 1.179)^***^	1.172 (1.149, 1.196)^***^	1.212 (1.114, 1.320)^***^

Model 1: unadjusted. Model 2: adjusted for age, sex, and ethnicity. Model 3: adjusted for model 2 plus place of household registration, education level, job type, family income per year, smoking status, alcohol intake status, habit of drinking tea, physical activity, SBP, DBP, HDL-CH, LDL-CH, TG, CHOL, CVD, and hyperlipemia.

95% CI, 95% confidence interval; OR, odds ratio; BAI, body adiposity index; WHR, waist-to-hip ratio; WHtR, waist-to-height ratio; BMI, body mass index; AIP, atherogenic index of plasma.

*p < 0.05; **p < 0.005; ***p < 0.001.

### 3.3 Restricted cubic spline curve fitting

**Figure 2:**
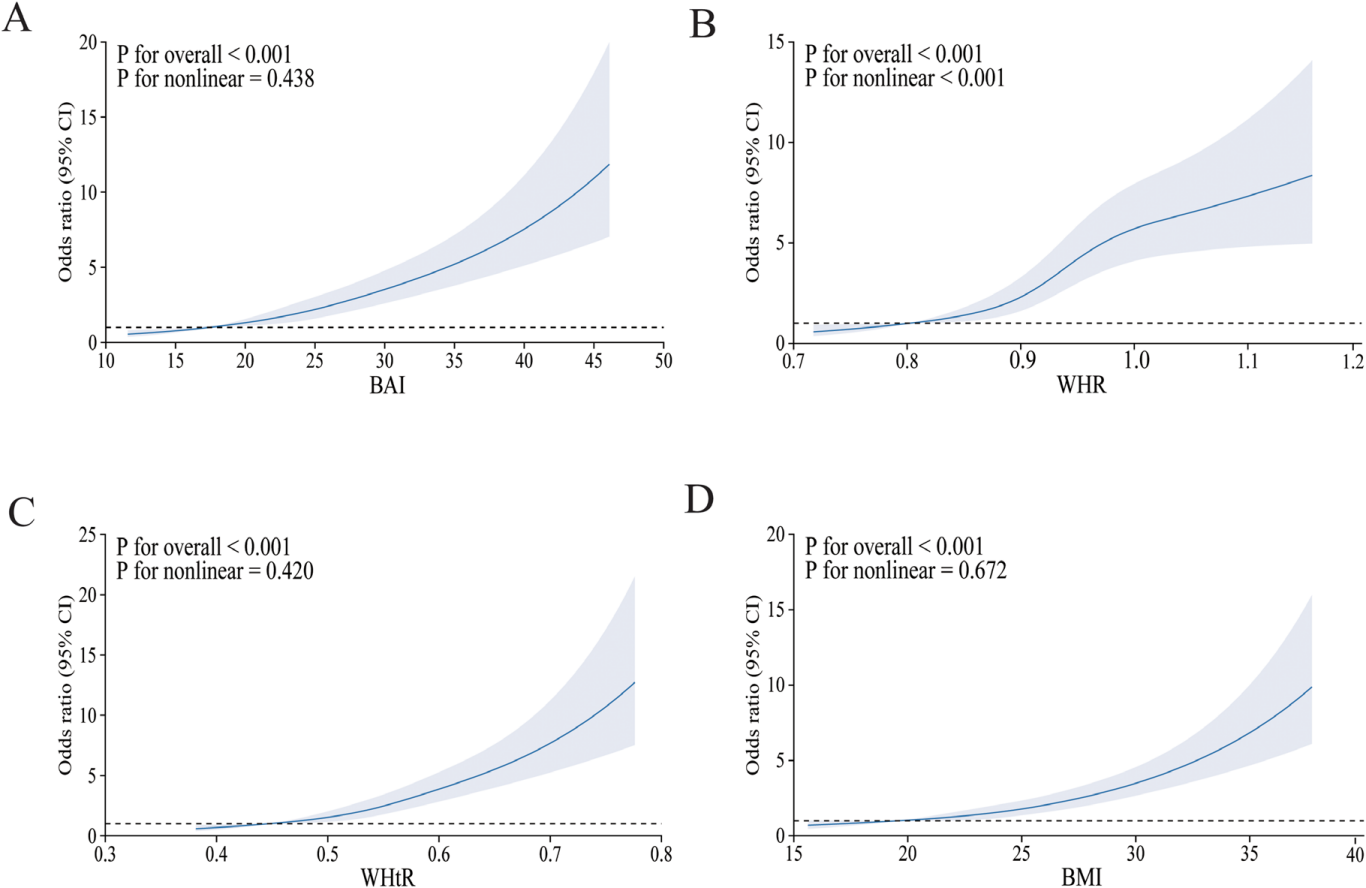
The RCS model of the association between obesity indices and risk of diabetes mellitus. OR, odds ratio; CI, confidence interval. **(A)** BAI; **(B)** WHR; **(C)** WHtR; **(D)** BMI. Adjusted for age, sex, ethnicity, place of household registration, education level, job type, family income per year, smoking, drinking, habit of drinking tea, physical activity, SBP, DBP, HDL-CH, LDL-CH, TG, CHOL, CVD, and hyperlipemia.

**Figure 2** depicts the relationship between four obesity indicators and diabetes mellitus using the RCS curve, with A–D depicting the dose–response relationship between BAI, WHR, WHtR, BMI, and diabetes mellitus after adjusting for all covariates, respectively. As the index increased, the incidence of diabetes mellitus gradually increased. Overall, there was a linear negative correlation between BAI, WHtR, BMI, and diabetes mellitus, which was particularly evident in BMI (non-linear *p* = 0.672). Furthermore, we could find a non-linear relationship between WHR and the prevalence of diabetes mellitus (non-linear *p* ≤ 0.001).

### 3.4 Mediation analysis

**Figure 3:**
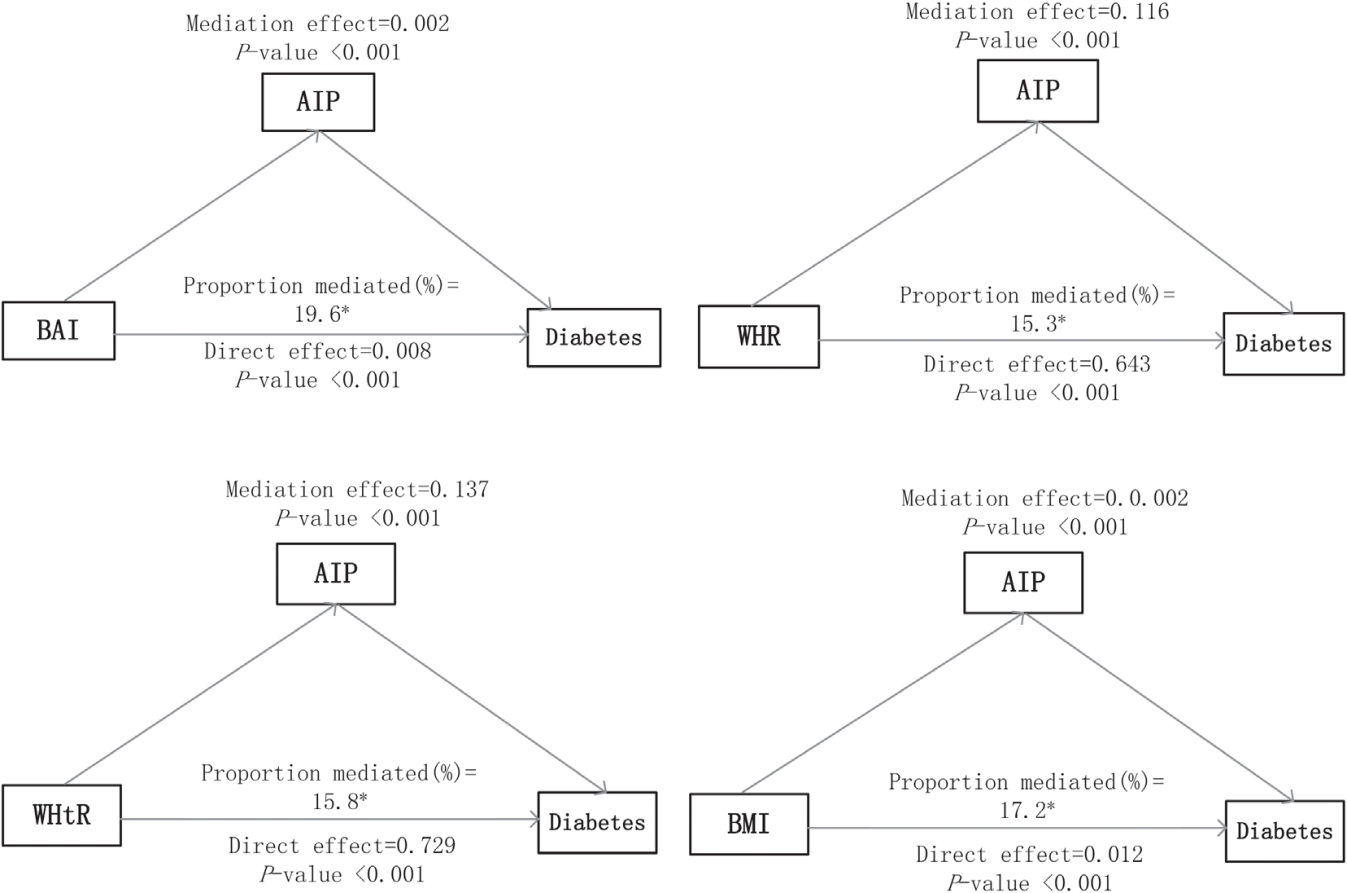
Mediation analyses of the AIP in the association of obesity indices with diabetes mellitus. The asterisk (*) indicates statistical significance, specifically p < 0.05.

**Figure 3** presents the direct and indirect effects of BAI, WHR, WHtR, and BMI on diabetes mellitus with AIP as mediators after adjusting all covariates. Overall, the AIP mediated the relationship between all four obesity indicators and diabetes mellitus. The findings suggest that the association of each obesity indicator with diabetes mellitus was mediated by the AIP, and the proportion of the total effect of obesity indicator mediated by the AIP was 19.6%, 15.3%, 15.8%, and 17.2% for BAI, WHR, WHtR, and BMI, respectively.

## 4 Discussion

This study aims to explore and elucidate the mediating role of the AIP in the relationship between multiple obesity indices (BMI, WHR, WHtR, and BAI) and the prevalence of diabetes in the hypertensive minority. The results reveal that the AIP significantly mediates the association between each of the four obesity indices and diabetes prevalence.

The co-occurrence of hypertension and diabetes has been well-documented in numerous studies (20, 21). For example, Liu et al. reported that the prevalence of diabetes among hypertensive outpatients could be as high as 24.3%, a rate significantly higher than that observed in the present study (22). This discrepancy may originate from differences in the diagnostic criteria for hypertension or differences in the study populations.

Previous studies have shown that the AIP, a marker of lipid metabolism, is significantly associated with diabetes (23). The AIP, reflecting the TG-to-HDL-C ratio, effectively captures dyslipidemia, a key factor in diabetes development (24, 25). Importantly, the role of the AIP is magnified in the context of hypertension, where dyslipidemia and insulin resistance frequently coexist.

Hypertension in patients is often accompanied by oxidative stress, which is known to play a significant role in the development of cardiovascular diseases (26). Oxidative stress can disrupt normal physiological functions, including lipid metabolism (27). The AIP acts as a mediator in multiple ways. An elevated AIP, resulting from abnormal lipid metabolism, can lead to increased formation of oxidized low-density lipoproteins (ox-LDL) (28, 29). These ox-LDL particles are taken up by macrophages, forming foam cells that contribute to atherosclerosis (30). The atherosclerotic process restricts blood flow to tissues, including the pancreas, impairing insulin secretion (31). Moreover, the AIP can induce chronic inflammation (32), with inflammatory cytokines interfering with insulin signaling pathways, leading to insulin resistance (33). In obese hypertensive patients, these mechanisms are further exacerbated, increasing the risk of diabetes onset (34).

BMI is a widely recognized measure of obesity, with a well-established link to diabetes (35, 36). A cohort study by Sattar et al. found a significant association between higher BMI and an increased risk of type 2 diabetes, particularly in obese individuals (37). Despite BMI’s inability to differentiate between fat and muscle, its simplicity and broad applicability make it a mainstay in clinical and epidemiological research (38). Studies also show a significant correlation between BMI and AIP, suggesting that BMI may influence diabetes prevalence by affecting lipid metabolism, with AIP as the mediating factor (39). WHR and WHtR, as indicators of abdominal obesity, have also been widely recognized for their association with diabetes (40). Zhu et al. highlighted that WHR and WHtR are positively correlated with type 2 diabetes risk, with WHtR being a superior predictor compared to BMI (41), and abdominal fat accumulation is a key contributor to insulin resistance, leading to diabetes (42).

The AIP is significantly associated with both WHR and WHtR, indicating that individuals with higher abdominal obesity tend to have elevated AIP levels, which may increase diabetes risk both directly and indirectly (43). The BAI, a newer obesity metric, has also been linked to diabetes risk, with research suggesting that it outperforms BMI in predicting body fat percentage (44). Studies show a significant correlation between BAI and AIP, highlighting the role of fat distribution in lipid metabolism (41). The relationship between hypertension and diabetes is complex, with hypertension not only increasing diabetes risk but also exacerbating the pathology of both conditions (45). This study found that elevated AIP significantly raises the risk of diabetes in hypertensive patients, likely due to hypertension-induced oxidative stress and inflammation impairing insulin signaling. Mediation analysis suggests that the AIP partially mediates the relationship between obesity indices and diabetes risk, implying that obesity may indirectly increase diabetes risk by elevating the AIP. These findings highlight the importance of early intervention in lipid metabolism for obese and hypertensive patients.

This study has several limitations. First, as a cross-sectional design, it cannot establish causality. Longitudinal studies are needed to confirm the causal relationship between the AIP and its mediating role between obesity and diabetes. Second, the sample is primarily composed of individuals from minority regions in southwest China, which may limit the generalizability of the findings. Future research should validate these results in more diverse populations. Additionally, only four obesity indices were examined; future studies should explore a broader range of obesity metrics and their associations with AIP and diabetes. One of the key strengths of this study is the use of a mediation analysis to explore the associations between obesity-related indices, AIP, and diabetes. Additionally, we conducted a comprehensive review of the existing literature, carefully considering and controlling for various potential confounding factors that may influence the relationship between obesity indices and diabetes. The application of a multivariable regression model further strengthens the accuracy and robustness of our conclusions.

## 5 Conclusion

In conclusion, the AIP plays a crucial mediating role in the relationship between obesity and diabetes risk in hypertensive patients. Elevated AIP levels, indicative of atherogenic dyslipidemia and insulin resistance, may partially explain the increased diabetes risk observed in this population. Addressing the AIP through targeted interventions may provide a novel approach to reducing diabetes incidence in hypertensive individuals with obesity. Further research is needed to elucidate the underlying mechanisms and develop effective strategies for managing this at-risk population.

## Data Availability

The data can be obtained upon reasonable request with the approval of the corresponding author. Requests to access the datasets should be directed to FH, hongfeng-73@163.com.
